# Supplementary material for: Long non-coding RNA LINC01234 regulates proliferation, migration and invasion via HIF-2α pathways in clear cell renal cell carcinoma cells
Source: PeerJ. 2020 Oct 14;8:e10149. doi: 10.7717/peerj.10149 (PMC7568479; doi:10.7717/peerj.10149)
Supplement: Supplemental Information 1 [file peerj-08-10149-s001.docx]

**Supplementary Table S1.**  RT-PCR primer sets used in this study.

**Primer sequences for RT-PCR**

**LINC01234**

5′-CAGGGTACCCCAAGCAAGTC-3′ (forward)

5′-GGGGTGAGAAGAGACAAGCG-3′ (reverse) .

**GAPDH**

5′-GTCAAGGCTGAGAACGGGAA-3′ (forward)

5′-AAATGAGCCCCAGCCTTCTC-3′ (reverse) .

**E-cadherin**

5′-AGTCACTGACACCAACGATAAT-3′ (forward)

5′-ATCGTTGTTCACTGGATTTGTG-3′ (reverse)

**N-Cadherin**

5′-CGATAAGGATCAACCCCATACA-3′ (forward)

5′-TTCAAAGTCGATTGGTTTGACC-3′ (reverse)

**HIF-2α**

5′-ATCAGCAAGTTCATGGGACTTA-3′ (forward)

5′-AAACCAGAGCCATTTTTGAGAC-3′ (reverse)

**VEGFA**

5′-ATCGAGTACATCTTCAAGCCAT-3′ (forward)

5′-GTGAGGTTTGATCCGCATAATC-3′ (reverse)
